# Supplementary material for: Plant-Root Exudate Analogues Influence Activity of the 1-Aminocyclopropane-1-Carboxylate (ACC) Deaminase Gene in Pseudomonas hormoni G20-18T
Source: Microorganisms. 2023 Oct 6;11(10):2504. doi: 10.3390/microorganisms11102504 (PMC10608949; doi:10.3390/microorganisms11102504)
Supplement: Supplementary file 1 [file microorganisms-11-02504-s001.zip › microorganisms-2600253-supplementary.pdf]

Supplementary Material

Plant-Root Exudate Analogues Influence Activity of the 1-Aminocyclopropane-1-Carboxylate (ACC) Deaminase Gene in *Pseudomonas hormoni* G20-18[T]

Ajay Madhusudan Sorty *, Fani Ntana †, Martin Hansen and Peter Stougaard *

Department of Environmental Science, Aarhus University, 4000 Roskilde, Denmark; fnt@bactolife.com (F.N.); martin.hansen@envs.au.dk (M.H.)

* Correspondence: amsorty@envs.au.dk (A.M.S.); pst@envs.au.dk (P.S.); Tel.: +45-52694976 (A.M.S.); +45-30231784 (P.S.)

† Present address: Bactolife A/S, DK2100 Copenhagen, Denmark.

Supplementary Figure S1

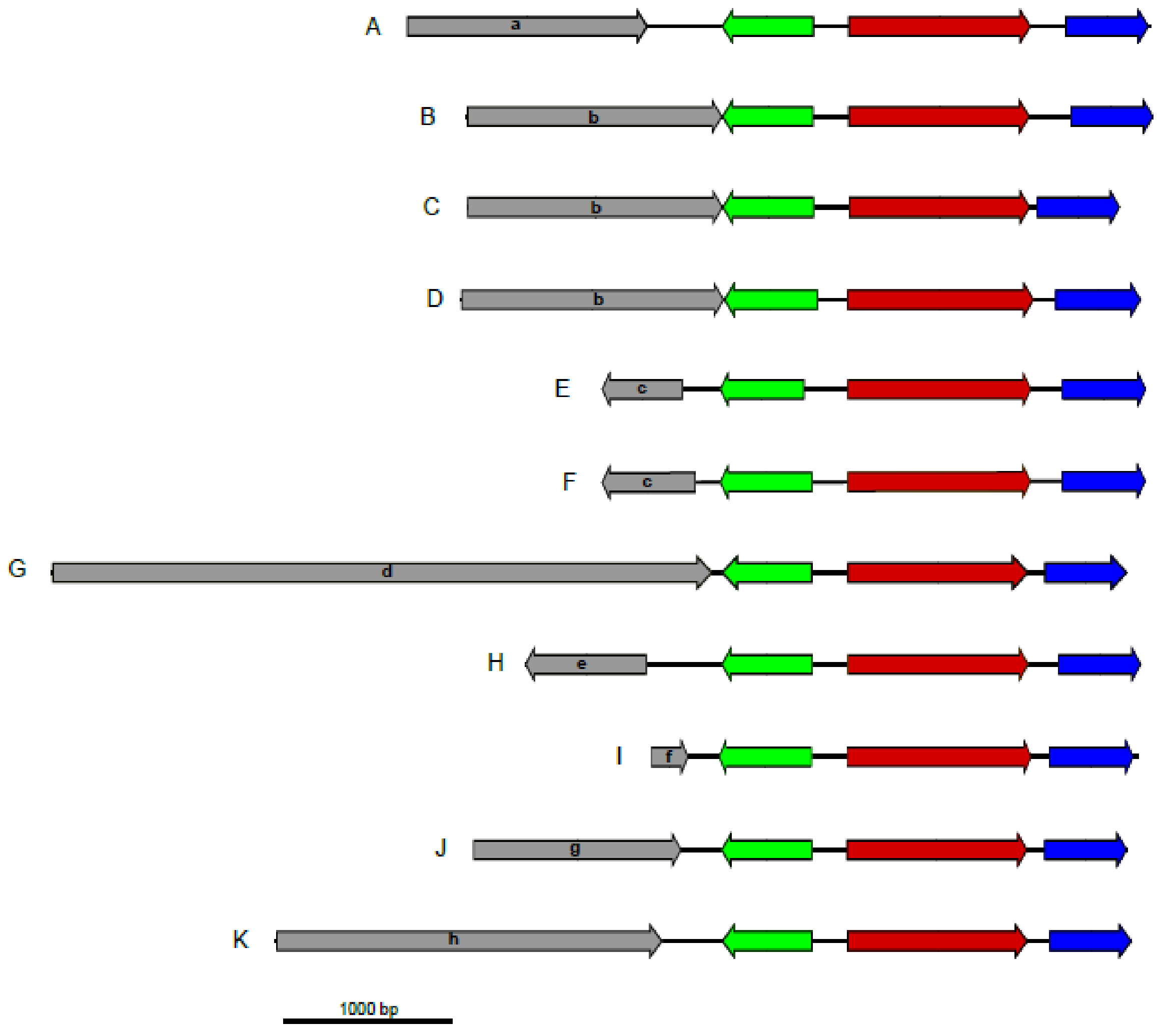

Pseudomonas acdR-acdS gene clusters with flanking genes. A, *Pseudomonas* sp. G20-18; B, *P. migulae* NBRC 102157; C, *P. fluorescens* NCIMB 11764; D, *Pseudomonas* sp. UW4; E, *P. ogarae* F113; F, *P. zarinae* SWRI108; G, *P. brassicacearum* 3Re2-7; H, *P. thivervalensis* BS3779; I, *P. fuscovaginae* UPB0736; J, *P. asplenii* B21-058; K, *P. palleroniana* Q1.
Color code: Green, leucine-responsive regulator, *acdR*; Red, ACC deaminase, *acdS*; Blue, transketolase; Grey, Micellaneous genes, a, sorbosone dehydrogenase family protein; b, c-di-GMP phosphodiesterase; c, putative membrane protein; d, response regulator; e, putative peptidoglycan binding domain-containing protein; f, alpha/beta hydrolase; g, glutathione-dependent formaldehyde dehydrogenase; h, topoisomerase DNA-binding C4 zinc finger domain-containing protein.

Supplementary Figure S1: *acdR* and *acdS* regions in *Pseudomonas hormoni* G20-18 with flanking genes as aligned with *acdR* and *acdS* regions in other PGPR strains.

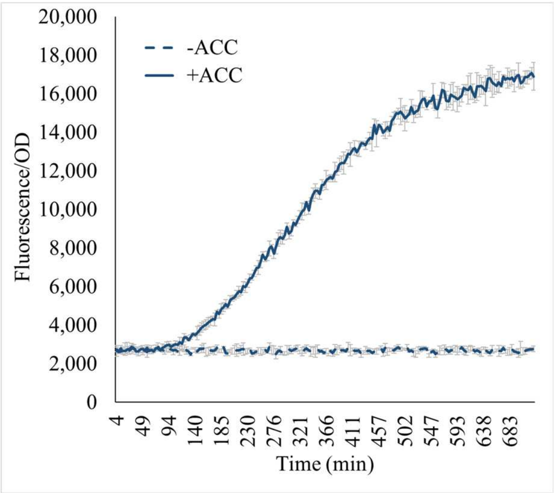

Supplementary Figure S2: Effect of ACC alone in M9 medium without glucose on the induction of *acdS* expression in *Pseudomonas hormoni* G20-18 deleted in *acdS* activity.

Supplementary Table S1: Wheat root exudate diversity as identified using LC-MS/MS (Excel file)

12-Hydroxydodecanoic acid CollisionEnergy:205060	LC-ESI	Orbitrap	Commercia	Trent Nort	JGI	M+H-H2O	199,169	0	1
12-Hydroxyjasmonate sulfate	LC-ESI	Orbitrap	Commercia	Ales Svatos	Riya Mene	M-H	305,07	0	1
12-Oxodihydrophytodienoic acid	LC-ESI	qTof	Isolated	Claudia Ma	Armando A	M+H-H2O	277,216	0	1
13-OxoODE	LC-ESI	qTof	Lysate	Dorrestein	lfnothias	M+H	-1	0	1
14-(hydroxymethyl)-5,9-dimethyltetracyclo[11.2.1.0�,�?	N/A	ESI-QFT	isolated	MoNA	MoNA:VF-I	[M+H]+	305,247	0	1
14-hydroxy-14-(hydroxymethyl)-5,9-dimethyltetracyclo[1	N/A	ESI-QFT	isolated	MoNA	MoNA:VF-I	[M+H]+	337,237	0	1
158031 - 40.0 eV	ESI	Orbitrap	Isolated	PI	Data Colle	M+H	190,086	0	1
15-Deoxy-delta-12,14-prostaglandin J2	LC-ESI	qTof	Commercia	Neha Garg	Nicole Aios	M+H	317,211	316,204	1
16-Hydroxyhexadecanoic acid CollisionEnergy:205060	LC-ESI	Orbitrap	Commercia	Trent Nort	JGI	M+H-H2O	255,232	0	1
1792831 - 30.0 eV	ESI	Orbitrap	Isolated	PI	Data Colle	M-H	285,207	0	1
17-Hydroxypregn-4-ene-3,20-dione - 30.0 eV	ESI	Orbitrap	Isolated	PI	Data Colle	M+H	331,227	0	1
1-AMINOADAMANTANE	ESI	qTof	isolated	MoNA	MoNA:Mo	M+H	152,143	0	1
1-AMINOCYCLOPROPANE-1-CARBOXYLIC ACID	ESI	Orbitrap	isolated	MoNA	MoNA:Mo	M+H	102,055	0	1
1-beta-D-Glucopyranosyl-L-tryptophan	LC-ESI	qTof	Isolated	Claudia Ma	Armando A	M+H	367,15	0	1
1h-indole-3-butanoic acid	ESI	Orbitrap	Commercia	BMDMS-N	BMDMS-N	[M+H]+	204,1	203,095	1
1-methyl-4-(6-methylhept-5-en-2-yl)-2,3-dioxabicyclo[2.2.	ESI	Orbitrap	Isolated	Wolfender	Luis Quiros	M+H	237,185	236,178	1
1-methyl-4-methylidene-7-(propan-2-yl)-1,2,3,3a,4,5,6,8a-	ESI	Orbitrap	Isolated	Wolfender	Luis Quiros	M-H2O+H	203,179	220,183	1
1-methyl-N-(2-(nicotinamido)ethyl)-1H-indole-3-carboxan	ESI	Orbitrap	Commercia	BMDMS-N	BMDMS-N	[M+H]+	323,15	322,143	1
1-METHYLNICOTINAMIDE	LC-ESI	Q-Exactive	Commercia	Alexandrov	Prasad	M+H	138,08	137,071	0
1-Naphthaleneacetic acid CollisionEnergy:102040	LC-ESI	Orbitrap	Commercia	Trent Nort	JGI	M-H	185,061	186,068	-1
1-OLEOYL-RAC-GLYCEROL	ESI	Orbitrap	isolated	MoNA	MoNA:Mo	M+H	357,3	0	1
1-PHENYLETHANOL	ESI	Orbitrap	Commercia	Dorrestein	Fernando V	M+H	123,08	122,073	1
2-(2-amino-4-hydroxy-6-methylpyrimidin-5-yl)acetic acid	ESI	Orbitrap	Commercia	BMDMS-N	BMDMS-N	[M+H]+	184,07	183,064	1
2-(2-furyl)-4-oxochromen-6-yl 3-bromobenzoate Collision	LC-ESI	Orbitrap	Commercia	Trent Nort	JGI	M-H	408,972	0	1
2-(3-hydroxy-5-methoxyphenoxy)-6-(hydroxymethyl)oxar	N/A	ESI-QFT	isolated	MoNA	MoNA:VF-I	[M-H]-	301,093	0	1
2-(3-oxobutyl)cyclopentane-1,3-dione	ESI	Orbitrap	Commercia	BMDMS-N	BMDMS-N	[M+H]+	169,09	168,079	1
2-(4-HYDROXYPHENYL)PROPIONIC ACID	ESI	qTof	isolated	MoNA	MoNA:Mo	M-H	165,056	0	1
2-(hydroxymethyl)-5-methoxy-4H-pyran-4-one	ESI	Orbitrap	Commercia	BMDMS-N	BMDMS-N	[M+H]+	157,05	156,042	1
2-(methylethylidene)butanedioic acid CollisionEnergy:102	LC-ESI	Orbitrap	Commercia	Trent Nort	JGI	M+H-H2O	141,055	0	1
